# Recent SARS-CoV-2 Outlook and Implications in a COVID-19 Vaccination Era

**DOI:** 10.1097/IM9.0000000000000072

**Published:** 2021-08-13

**Authors:** Teddy Ehianeta, Said Abdulrahman Salim Mzee, Muslimat Kehinde Adebisi, Oluwayemisi Ehianeta

**Affiliations:** 1 Institute of Biological Chemistry, “Academia Sinica,” Taipei, Taiwan, China; 2 Jiangbing Hospital, Affiliated to Jiangsu University, Zhenjiang, Jiangsu, China; 3 University of Windsor, Windsor, Ontario, Canada; 4 Taiwan University of Science and Technology, Taipei, Taiwan, China.

**Keywords:** breakthrough infections, COVID-19, EUA, immune response, SARS-CoV-2, vaccine

## Abstract

While repurposed drugs came in handy earlier in the wake of the coronavirus disease 2019 (COVID-19) pandemic, vaccination has been considered a more sustainable approach. The recent spikes have been linked to “double,” “triple,” and even multi-mutant variants, thus renewing calls for deeper structural and functional insights of severe acute respiratory syndrome coronavirus 2 (SARS-CoV-2) as a lead to rationale design of therapeutics, vaccines, and point-of-care diagnostics. There is a repertoire of findings from the earliest SARS-CoV-2 molecular mimicry to evade host immunity cum host immune responses to the role of the viral glycocalyx in modulating the susceptibility and severity of infection through attraction and repulsive interactions. Recently, molecular studies of some viral components that aid infection in the face of vaccination seem unending. In addition, the wave of infections and the attendant case fatality ratios have necessitated the need for emergency use authorizations for COVID-19 vaccines and in vitro diagnostics. This review provides key updates of SARS-CoV-2, current antigenic and formulation strategies, with emergency use authorizations considerations for future vaccine candidates and diagnostics. We also premise that despite the difficulty in modeling and analyzing glycans, understanding and exploiting their roles in the SARS-CoV-2 architecture is fundamental to glycan-based COVID-19 vaccines devoid of inconsistent clinical outcomes.

## Introduction

The beginning of the decade ushered in the outbreak of the novel coronavirus disease 2019 (COVID-19)^1^ – a public health concern of global significance that transitioned quickly from an epidemic to a global pandemic within a few weeks. Although the socio-economic and geopolitical impacts of the pandemic are notable, the public health concerns remain at the core. By June 2021, over 180 million confirmed cases with an approximate 2.2% mortality rate have been reported according to a World Health Organization report (https://covid19.who.int/). This report also depicted the Western Pacific and Americas to bear the least and highest burden of COVID-19, respectively. Typically, the clinical manifestations of the disease are fever, cough, dyspnea, malaise, fatigue and sputum/secretion,^2^ albeit prolonged pulmonary, neurological, and cardiological consequences have also been seen in numerous cases.^3^ Currently, there is no standard treatment for the disease; however, the earliest interventions involved the use of repurposed drugs ranging from RNA-dependent RNA polymerase inhibitors,^4^ protease inhibitors,^5^ and immunomodulatory drugs^6^ to miscellaneous agents such as lipoglycopeptides,^7^ Ivermectin,^8^ and Tocilizumab. Despite the promising clinical outcomes of these therapeutics, a few events have tempered their use and approval, exemplified by little or unfavorable pharmacokinetic profiles, systemic toxicities, lack of randomized trials, small size of the cohort, and in some cases, inconsistencies in the data from case series or clinical trials.

In parallel, vaccination has been considered a reliable route to post-pandemic normalcy, as demonstrated by vaccines currently in use and others in preclinical and late-clinical stages, all at accelerated pace. The urgency of the demand has also necessitated the emergency use authorization (EUA) of some of these interventions. Fundamentally, development of a vaccine against a viral strain or multiple strains is premised on the understanding of host immune responses to infections. Pointedly, some studies have established strong correlations between vaccine-elicited neutralizing antibodies and a reduction of viral loads in non-human primates infected with severe acute respiratory syndrome coronavirus 2 (SARS-CoV-2).^9–12^ Indeed, the causative organism of COVID-19, SARS-CoV-2,^13^ is expectedly profiled as a lead to developing effective therapeutic and prophylactic agents; however, certain structural features remain elusive in the face of a surge in infections. To this end, this review provides key updates on SARS-CoV-2, especially the under-reported place of glycans in the pathogenesis of the disease, and a synopsis of literature on the host responses to immunogenic components of the vaccine candidates. By keeping up with these studies, we aim to provide a basis for broader and informed rational design of anti-COVID-19 agents, while using key EUA submissions as a watchdog for the ideal vaccine candidate.

## Structural profile of SARS-CoV-2

As a requisite concept, the structural and biochemical profile of SARS-CoV-2 underpins the pattern of transmission, pathogenesis of COVID-19, and rational development of drugs and vaccines. The coronaviruses (CoVs) are lipid-enveloped single-stranded positive-sense RNA viruses responsible for multiple respiratory disorders of varying severity in humans. Besides representing the largest RNA viruses, another typical feature of all CoVs is their ability to encode the nucleocapsid (N) protein, the structural spike (S) glycoprotein, and two smaller proteins – the envelope (E) protein and the membrane (M) glycoprotein.^14^A report by Lu et al. on genomic profiling of the *Betacoronavirus* SARS-CoV-2 established the genomic size to be in the range of 29 kb, with a phylogenetic analysis that showed that SARS-CoV-2 belonged to the subgenus *Sarbecovirus*, displaying higher similarity to bat-SL-CoVZC45 and bat-SL-CoVZXC21 than to the human SARS-CoV.^15^ In addition, Cryo-electron microscopy (Cryo-EM) images elucidated the *Betacoronaviruses* to be spherical particulate matter with a bordering lipid bilayer that encapsulates an opaque viroplasm internally and protruding S trimers externally.^16,17^ Contextually, the transmembrane S glycoproteins, representing the antigenic machinery decorated as homotrimers elaborated on the viral surface of CoVs, facilitate viral entry into host cells^18^ through unique receptor sites such as the human dipeptidyl-peptidase 4 receptor for the Middle East respiratory syndrome coronavirus S,^19^ the angiotensin-converting enzyme 2 (ACE2) for the severe acute respiratory syndrome (SARS) coronavirus S^20^ and SARS-CoV-2 S.^21^ This event informs a highly conserved significant structural change to the post-fusion form from the pre-fusion conformation of the S protein. In addition to the N, S, E, and M proteins, sixteen non-structural proteins (NSP1-NSP16) have also been profiled and by aid of computational studies, NSPs 3, 5, 11, 14, and 15 were found to be ideal targets for three drug candidates (DB01977, BD07132, and DB07535).^22^ Despite the paucity of data on NSPs as targets for anti-COVID-19 agents, these candidates strongly support the need for a multidirectional approach to curb the effects of SARS-CoV-2.

### Spike (S) protein

**Figure 1:**
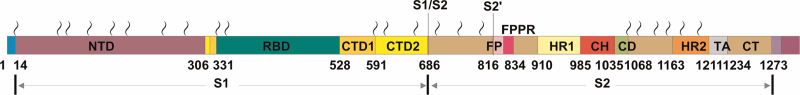
Schematic representation of the full-length SARS-CoV-2 S protein showing key subunits of S1 and S2, with wavy symbols as glycans. SARS-CoV-2: severe acute respiratory syndrome coronavirus 2.

**Figure 2:**
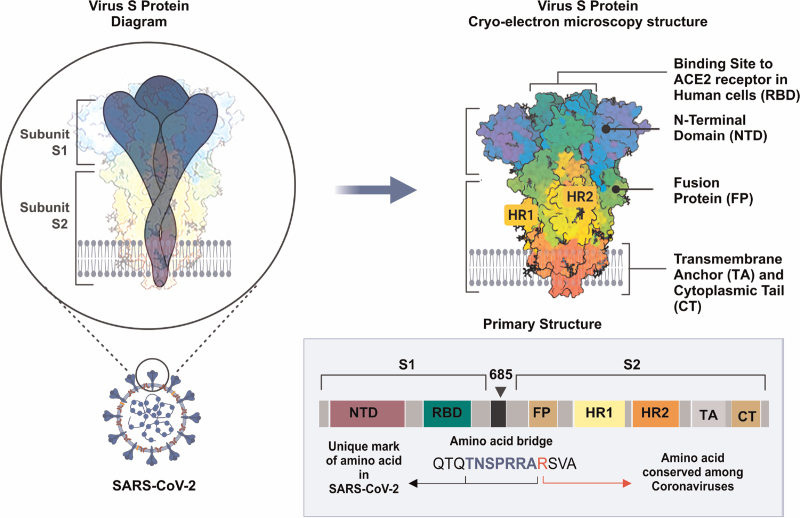
**Detailed schematic representation of the SARS-CoV-2 S protein regardless of conformation state**. ACE2: angiotensin-converting enzyme type 2; SARS-CoV-2: severe acute respiratory syndrome coronavirus 2.

The protein component of the S glycoprotein is depicted as a single polypeptide chain precursor of approximately 1300 amino acids, which can be cleaved by the host furin-like proteases into an amino (N)-terminal S1 subunit and a carboxyl (C)-terminal S2 subunit^23,24^ – the two structural subunits of the S glycoprotein. Expectedly bifunctional, the subunits impact both receptor binding and fusogenic functions. The S1 subunit is responsible for adhering to host cell receptors while the S2 ensures the fusion of the host and viral cellular membranes. The distal S1 folds as four independent domains comprising the N-terminal domain, two C-terminal domains, and the receptor-binding domain (Figure 1)^25,26^ that houses the receptor-binding motif that selectively binds to the ACE2 receptor of the host.^27^ As shown in Figure 2, the S2 subunit is made up of the fusion peptide (FP), heptad repeat 1 and 2 (HR1/HR2), transmembrane anchor (TA), and the intracellular tail or cytoplasmic tail (CT). It is noteworthy that in Figure 2 the extracellular domain does not bear the heptad repeat 2 region and the transmembrane anchor. Two proteolytic sites believed to be key to the viral infection process are the S1/S2 protease cleavage site and the FP site. The S1/S2 interphase features the novel furin cleavage site reported to be the site of action for protease inhibitors^28^ such as transmembrane serine protease 2 inhibitors (eg, Camostat mesylate) whereas the FP cleavage site primes the host membrane for fusion upon penetration.^29^ Zhou et al. captured a snapshot of the interaction between S and human ACE2 using Cryo-EM,^30^ a fundamental that enhances our understanding of how the virus engages the S protein to gain entrance into a host cell. Similar studies are all geared to interfering with the Spike-ACE2 interaction. Despite this feat, protein structures elucidated by Cryo-EM or X-ray crystallographic assays often exclude the sugar components (glycans) that cover large bands of the S surfaces.

### Glycans and their essentiality in SARS-CoV-2

Since the S protein is the main target of neutralizing antibodies, efforts geared towards vaccine and drug development center around the extensively glycosylated S protein. There are heightened conversations among structural biologists and glycobiologists about the structure elucidation of the glycan (polymers of sugar residues) architecture on the S protein as a strategy to enhance anti-COVID-19 interventions, whether as prophylactics or therapeutics. The current evidential basis relating to glycosylation of viral proteins depends on glycan processing enzymes within the virus or the host. These glycans are also key players in the propagation sequence of the virus, for one, the masking of the S protein from the host antibody machinery. The complexity of the SARS-CoV-2 biology is likely to be connected to the delay in developing safe anti-viral interventions, especially considering the emergency and pandemic status of COVID-19. Indeed, glycosylation within the microbial milieu has far-reaching implications due to glycan roles such as mediating protein stability, folding, antigenicity, immunogenicity, and intermolecular interactions.^21,31^ These roles appear to be ignored in the current discussion of SARS-CoV-2 due to difficulties in modeling the complex glycan network and poor sensitivity of analytical instruments, despite being a major composition on the glycoprotein scaffold. For instance, according to Crispin's model of the glycan framework on the viral S protein, each trimer displays 66 *N*-linked glycosylation sites (22 *N*-linked glycan sequons per protomer).^32^ Crispin et al. reported that the higher the mannose content is, the less the glycans resemble the host cell glycans and the less camouflaged the surface S protein is from the host's immune system. It is now well documented that the S protein utilizes this framework to evade the host humoral immune system by molecular mimicry, thus shielding the immunogenic protein epitopes.^33–35^ This glycosylation profile has been strongly posited to likely influence the clinical outcomes in the vaccination campaign, especially with the advent of mutant strains.

Stemming from the unreliability of X-ray crystallography and Cryo-EM techniques to spot glycosylated portions of the SARS-CoV-2 framework, the Crispin group adopted a site-specific mass spectrometric approach to reveal all monomeric *N*-linked glycan sequons per protomer of the S protein. Similar results were obtained by Yang's group,^36^ which used a stepped collision energy mass spectrometry approach that revealed the *N*-glycosylation outline of the viral S proteins along with the glycan composition and site-specific numbers of glycans. In parallel, Azadi et al. engaged the proficiency of high-resolution liquid chromatography-tandem mass spectrometry to identify not only 17 out of the predicted 22 *N*-linked glycans sites, but serendipitously also two *O*-linked glycans out of the four predicted sites.^37^ These slight differences in methodologies and results among the glycan mappings allude to the challenges in consistently producing a glycosylated spike protein for vaccine development owing to the diversity in glycosylation which in turn depends on the preparatory conditions of the protein.

CoVs typically engage certain domains on the S protein that can interact with different glycoconjugates as attachment factors or secondary entry receptors. Esko et al. reported that S protein binding depends on ACE2 and heparan sulfate.^38^ Using docking studies and in vitro assays, they submitted that (i) by initial binding to heparan sulfate, the spike's receptor-binding domain adoption of an open conformation enhances S-ACE2 interactions; (ii) the eventual SARS-CoV-2 infection is interdependent on heparan sulfate and ACE2; and (iii) based on these correlations, modification of host heparan sulfate can downregulate SARS-CoV-2 infections. This submission aligns with that of Cai et al., which in elucidating the pre-fusion and post-fusion conformations of the S protein, posited that the unconstrained transition from the former to the later conformation is independent of ACE2-rich cells. In addition, the post-fusion conformation with its stable and rigid structure is aided by strategically decorated *N*-linked glycans to serve functions other than membrane fusion.^39^ In this context, the glycan architecture serves as a gate that shields and controls the opening of the spike protein – a basic requisite for infections.

## Host immune responses in COVID-19

Although host immunity is an essential protection against the disease, precise regulation of innate immunity is rate-limiting to the progression of immunopathogenesis.^40^ Since the pandemic, it is estimated that asymptomatic individuals and symptomatic without life-threatening conditions account for 40%–80% of the infected population,^41^ while 15% account for patients with infections that progress to severe pneumonia and 5% show signs of acute respiratory distress syndrome, septic shock and multiple organ failure in some cases.^42^ The degree of severity is doubtless related to the role of host immunity, which might be connected to genetic variations beside factors such as pre-existing medical conditions, age, and sex. Bearing the number of available reports, our understanding of immunogenomics in infected patients is still evolving. For instance, in strengthening the genetic basis of COVID-19 heterogeneity, emerging body of evidence has linked ABO blood groups^43^ and ACE2 receptor polymorphisms^44^ to COVID-19 susceptibility. In addition, Leite et al. reported preliminary evidence that linked immunogenetic markers [human leukocyte antigen B (HLA-B) and interleukins (IL-6, IL-10, and IL-12B)] to death by COVID-19.^45^ These correlations, although debatable, can only be subject to further investigations in immunogenomic studies.

Substantially, there are unique clinical and biochemical characteristics obtained from patients that depend on the severity of the disease. As a case in point, Chakraborty et al. reported proinflammatory IgG Fc structures, including afucosylated IgG1 as key features in grading the severity of inflammation in infected individuals.^46^ Since the virus principally attacks ACE2-abundant regions such as the respiratory tract richly decorated with alveolar type 2 cells,^47,48^ the S-protein is primed by cellular surface proteolytic enzymes, stimulating viral and cellular membrane fusion. The viral neutralization process proceeds with toll-like receptors recognizing the viral RNA, phagocytosis of the virus, and simplification of the structural components. The phagocytes (antigen-presenting cells), such as dendritic cells, migrate to lymphatic organs and display major histocompatibility complex class 1 and 2 molecules to T-cells via T-cell receptors. This influences the defense pathway of T-cells to the viral entry. In parallel, CD-8 cytotoxic T-cells secrete cytokines such as IL-6 that clear infected cells from the host. The CD-4 T helper cells, on the other hand, enhance cytotoxic T-cells influencing B-lymphocyte production.^49^ This mechanism is crucial for the viral opsonization period and during the post-vaccination period. Once the T-cells activate the B-cells, B-lymphocytes mature and replicate, creating clones of plasma cells which produce antibodies that recognize and neutralize the virus.

## COVID-19 vaccines: formulation strategies and prospects

Exploiting the host translation mechanism, viruses insert their genomic material, thus influencing viral replication, albeit the duration of antibody responses to viral entry is essential to minimizing the damage caused by SARS-CoV-2 infections.^50^ To enhance the rate of viral recognition and neutralization induced by vaccination, the concept of designing a “wholistic antigenic” vaccine that highlights incorporating the M and N proteins in a single vaccine formulation^51^ might be an eventual prospect for COVID-19 and future pandemics. In view of other strategies, DNA and mRNA vaccines although aim to serve the same purpose, they differ in the following sense: (i) while mRNA is processed directly in the cytosol, DNA is required in the nucleus to be decoded; (ii) unlike DNA vaccines, the mRNA vaccines are safer since they are not integrated with host genome thus lack long-term effect on the host's genetic pool; (iii) mRNA tends to denature post protein formation, impacting a short half-life. Despite its immunogenicity, the setback of the mRNA vaccine is its instability and inefficient in vivo delivery.^52^ In addition, to prevent host RNase-mediated degradation before reaching the target site, the vaccines are encapsulated in positively charged lipid nanoparticles as carrier, self-assembling into virus-sized particles that can be administered through different routes.^53^ Nanotechnology has found possible benefits in the host immunity conditional to size, surface charge, shape, and hydrophobicity in addition to logistic factors. Albeit nanoparticles can activate the immune responses and diminish cytokine toxicity, but they are principally of merit in targeted site-specificity, prompting a resilient immune reaction. Typically, the lipid nature of nanoparticles enhances entry of genetic material through the lipid cell membrane^49^; however, the current mRNA vaccines are encapsulated with lipid nanoparticles that require long-term sub-zero storage conditions (−20 to −80°C).^54,55^ This feature poses a logistical challenge for low-income communities and consequently the global objective of flattening the curve to the barest minimum. In a similar vein, a viable single-dose vaccination strategy was also reported, which engaged spike-functionalized ferritin nanoparticles that elicited an enhanced antibody response against lentiviral SARS-CoV-2 pseudovirus treatment. These functionalized S proteins were obtained by fusing either the full length S ectodomain (residues 1-1213) or the C-terminal free S protein (residues 1-1143) to the *Helicobacter pylori* ferritin as an immunoadjuvant.^56^

## SARS-CoV-2 vaccine targets

### Vaccines and concerns in the development of SARS-CoV-2 vaccines

Vaccines generally trigger an immune response to recognize and build up defences against specific pathogens but there are a few concerns associated with novel vaccines during a public health emergency. Understanding vaccine-induced immunopathology is key to designing effective vaccines for emerging infectious diseases. Like other vaccines, the major concern with SARS-CoV-2 vaccines is the safety profile in a healthy population, therefore the duo considerations of (i) the vaccine should not elicit life threatening adverse effects in patients, and (ii) the vaccine should prevent severe diseases in immunized persons. Pointedly, the efficacy of the COVID-19 vaccine in protecting against multiple/heterologous viral variants is of high importance, especially with the recent emergence of “double” and “triple” mutant strains in different parts of the world. There are views that current vaccines being used under the EUA have been found to be less effective against some newer strains, thereby restricting their use in localities plagued by those latest strains.^57^ Potential vaccines are also expected to produce robust immunological responses at administered concentrations especially in a population at the highest risk of infections, that is, the elderly and immunocompromised.

The rapid structural elucidation of the SARS-CoV-2 virus, which began in early 2020, aimed at identifying possible targets for vaccine development, and resulted in vaccines generating neutralizing antibodies with adequate protective titers to prevent disease enhancement upon subsequent infections. However, vaccine induction of enhanced diseases could be antibody-mediated, resulting in antibody dependent enhancement (ADE) and vaccine associated enhanced respiratory disease (VAERD), or could be mediated by Th2 cell-based immunopathologic responses causing VAERD.^58,59^ Immunogenic response-triggered antibodies which bind to viral antigens but fail to effectively neutralize the virus can cause enhanced diseases through the enhancement of viral replication or the formation of immune complexes that activate inflammation pathways.^60^ This is usually the case with ADE when the antibodies triggered by immunogenic responses complex with viral antigens but fail to effectively neutralize the virus due mainly to suboptimal concentrations and inaccurate specificity amongst other reasons,^58^ thereby enhancing viral replication and facilitating viral infection of cells.^60,61^ ADE was first reported with dengue virus (DENV), which has 4 known serotypes. The immune response to a specific DENV serotype can produce neutralizing antibodies which are effective long term against homologous variants and are cross-reactive with heterologous DENV serotypes.^62,63^ These cross-reactive antibodies are protective against heterologous serotypes for only a few months before the concentrations drop to suboptimal levels. At this point, the risk of severe dengue symptoms after infection with heterologous DENV is higher in such individuals compared to naive individuals. In the context of COVID-19, it is not unlikely, although not evidential yet, to correlate the sudden spike in infections and mortality (second and third wave) to a cross-reactivity between the native SARS-CoV-2 and the newer variants.

While ADE is not specific to respiratory diseases, VAERD refers to clinical symptoms of severe respiratory viral infections due to prior immunological responses induced by vaccination. It was first reported after markedly worse respiratory diseases were observed in study participants of the formalin-inactivated respiratory syncytial virus vaccine group and not in the placebo group.^60^ This was reported as the cause of inflammation and airway obstruction in some participants of the clinical trials who were immunized with the formalin-inactivated respiratory syncytial virus vaccine.^64^ VAERD is also mediated by Th2-biased immune responses, which cause allergic inflammation through cytokine production and eosinophilic infiltration, resulting in aggravated airway dysfunction and delayed viral clearance.^63^ Potential concerns of ADE and VAERD following the use of SARS-CoV-2 vaccines have arisen from animal models used in assessing safety and efficacy of some SARS-CoV vaccines after enhanced diseases were observed in some animals following viral challenge,^65–68^ but the efficacy data of phase 3 trials of current EUA SARS-CoV-2 vaccines do not report enhanced diseases in the months following the use of the vaccines.^69,70^ Recent clinical trials have also shown the reduced effectiveness of some EUA vaccines against some newer variants of SARS-CoV-2, especially B.1.351; however, ADE and VAERD upon infection with these newer strains of the virus have not been reported^57^ as at the time of this review. Although concerns associated with vaccine-induced disease enhancement have been allayed, the effect of declining antibody titers after vaccination or infections and the potential long-term safety signals remain unknown since COVID-19 vaccines, including the EUA vaccines, still require the Food and Drug Administration (FDA) approval.

### COVID-19 vaccine: antigenic strategies

Vaccines currently in use are categorized based on the nature of the antigen used in their production, which also determines the storage and administration protocol. The representative antigenic strategies in use or trialed for development against the SARS-CoV-2 are presented in Table 1.

**Table 1:** Representative vaccine candidates in clinical development with those granted EUA approvals.

Vaccine type	Licensed vaccines	COVID-19 vaccine candidates in Phase 3 and 4 clinical trials	Developer
Live attenuated vaccine	Oral polio vaccine, measles vaccine, yellow fever, rotavirus, mumps, influenza, rubella, varicella, tuberculosis	COVI-VAC (Phase 1)^∗^NCT04619628	Codagenix Inc/Serum Institute of India
Inactivated vaccine	Inactivated polio vaccine, pertussis, hepatitis A, rabies	CoronaVac, SARS-CoV-2 vaccine (inactivated)NCT04756830 (Phase 4)NCT04747821 (Phase 4)	Sinovac
		Inactivated SARS-CoV-2 vaccine(Vero cell)NCT04510207 (Phase 3)NCT04612972 (Phase 3)	Sinopharm + China National Biotech Group + Wuhan Institute of Biological Products + Beijing Institute of Natural Products
		SARS-CoV-2 vaccine (vero cells)NCT04659239 (Phase 3)	Institute of Medical Biology + Chinese Academy of Medical Sciences
		QazCovid-in-COVID-19 inactivated vaccineNCT04691908 (Phase 3)	Research Institute for Biological Safety Problems, the Republic of Kazakhstan
		Whole-Virion inactivated SARS-CoV-2 vaccine (BBV152)NCT04641481 (Phase 3)	Bharat Biotech International
Protein subunit	Hepatitis B, *Haemophilus influenzae* B, pertussis, human papillomavirus, pneumococcal bacteria, meningococcal bacteria	SARS-CoV-2 rS/Matrix M-1 adjuvant (Full length recombinant SARS-CoV-2 glycoprotein nanoparticle vaccine adjuvanted with Matrix M)NCT04611802 (Phase 3)NCT04583995 (Phase 3)	Novavax
		Recombinant SARS-CoV-2 vaccine (CHO cell)NCT04646590 (Phase 3)	Anhui Zhifei Longcom Biopharmaceutical+ Institute of Microbiology, Chinese Academy of Sciences
RNA based vaccine	None	mRNA-1273NCT04470427 (Phase 3)NCT04760132 (Phase 4)	Moderna + Institute of Allergy and Infectious Diseases (NIAID)
		BNT162 (3 LNP-mRNAs)NCT04368728 (Phase 3)NCT04713553 (Phase 3)NCT04760132 (Phase 4)NCT04780659 (Phase 4)	Pfizer/BioNTech + Fosun Pharma
		CVnCoV vaccineNCT04652102 (Phase 2/3)NCT04674189 (Phase 3)	CureVac AG (Germany)
Viral vector(Non-replicating)	Ebola	ChAdOx1-S (AZD1222)NCT04536051 (Phase 3)NCT04516746 (Phase 3)NCT04760132 (Phase 4)	AstraZeneca + University of Oxford
		Recombinant novel coronavirus vaccine (Adenovirus type 5 vector)NCT04526990 (Phase 3)	CanSino Biologics + Beijing Institute of Biotechnology
		Gam-COVID-Vac Adeno-based (rAd26-S+rAd5-S)NCT04560396 (Phase 3)NCT04741061 (Phase 3)	Gamaleya Research Institute + Health Ministry of the Russian Federation
		Ad26.COV2.SNCT0405722 (Phase 3)	Janssen Pharmaceutical

WHO DRAFT landscape of COVID-19 candidate vaccines, 2020 (https://www.who.int/publications/m/item/draft-landscape-of-COVID-19-candidate-vaccines) Accessed on April 4th, 2021. COVID-19: coronavirus disease 2019; EUA: emergency use authorization; SARS-CoV-2: severe acute respiratory syndrome coronavirus 2. ^∗^ We envisage that with time other vaccine candidates will be released into clinical trials, this list represents a representative list as at the date enlisted.

Live attenuated vaccines are prepared from whole virions that have been weakened through repeated passages in a cell culture until virulence is reduced and pathogenicity is lost. This type of vaccine thus induces a mild infection akin to the native infection, leading to a strong immune response and long-term immunity, a desirable feature in vaccine candidates. However, there are safety concerns with the use of attenuated vaccines due to their potential to revert to virulent forms, exacerbating the disease conditions, especially in immunocompromised individuals.^71^ Our search indicated that there is only one live attenuated vaccine currently in the early phase of clinical trials. Whole inactivated vaccines, on the other hand, contain viruses that have been killed through chemical or physical processes, reducing the risk of causing diseases and making these vaccines more stable. They are, however, generally less immunogenic and may require multiple doses or an immunoadjuvant to elicit sufficient immune responses since they do not replicate.^72^

Protein subunit vaccines host the antigenic portions of the pathogen responsible for eliciting a protective immune response. These antigens are purified from the virus and devoid of live components of the pathogen. Therefore, formulations of this type of vaccines require the inclusion of an adjuvant to stimulate stronger immune responses and produce enhanced immunological memories. Smith et al. recently disclosed the development of a SARS-CoV-2 subunit vaccine (NVX-CoV2373) from stable pre-fusion S protein with immunoadjuvant Matrix-M (NCT04368988) using mice and baboons as test subjects.^73^ Their results not only are consistent with ongoing phase 1 and 2 clinical trials but also highlight the low dose-induced immunogenicity, thermostability, and high binding affinity of NVX-CoV2373 nanoparticles to ACE2. Although the histopathological assessment of test subjects indicated no evidence of VAERD, it is our view that extensive blood coagulation studies ought to be carried out alongside the findings. This centers on claims and counterclaims of blood clots with associated mortalities in individuals inoculated with the current EUA-approved vaccines. Virus-like particle vaccines also employ the technology of subunit vaccines but present many copies of the antigen in a three-dimensional virus-like structure, so the antigens may be more immunogenic and not require the addition of adjuvants.^74^ New generation of vaccines include but not limited to recombinant viral vector vaccines and nucleic acid vaccines. They are developed using a classic technology where specific antigens from a pathogen are isolated and advanced vaccine platforms are used to make the antigens elicit immune responses, thus providing a rapid vaccine development opportunity as well as a better safety profile because of its specificity.

Viral vector vaccines contain a viral vector that has been genetically modified to reduce its virulence; the vector carries the genetic code of antigens of the target pathogen, which have been copied using recombinant DNA methods. Recombinant viral-vectored vaccines mimic natural infections with the vector to elicit targeted immune responses against the genetically encoded viral antigen. Vector vaccines can be replicating or non-replicating. Replicating vector vaccines infect the cells where vaccine antigens are produced and replicate the viral vector to infect new cells, consequently producing more antigens. On the other hand, the non-replicating vector vaccines access the cells to produce vaccine antigens without replicating new virus particles. Since antigens are produced within the host, both humoral and cellular immune responses are stimulated, raising the possibility of a single dose to achieve adequate protection,^75^ although most of the viral vector vaccines that are in clinical trials require multiple doses. Human and chimpanzee adenoviruses have been used as sources for viral vectors to elicit good immunologic responses; however, there are concerns about pre-existing immunity against the human adenovirus, which reduces its immunogenicity relative to the chimpanzee adenovirus that has lower seroprevalence in most populations.^76^

In nucleic acid vaccines, specific antigens are isolated and delivered as DNA or RNA constructs encoding the viral antigens. As the nucleic acid accesses the cell, it initiates the synthesis of the antigenic proteins, which in turn elicits immune responses like those produced by natural infections.^74^ DNA vaccines require electroporation following injection for effective cellular uptake and must still be transcribed to mRNA before uptake,^72^ while mRNA vaccines, on the other hand, are quite unstable and readily removed by host cells upon injection. To this end, delivery techniques, which modify the nucleosides-like encapsulation of RNA in liposomes, have been developed to prevent degradation of the vaccine. Lipid nanoparticles are the carrier molecules often used in aiding the uptake of mRNA into cells immediately after injection.^77^ Although nucleic acid-based vaccines induce both humoral and cellular immune responses, multiple doses are still required for both DNA and RNA vaccines to provide long-term immunity. There are several nucleoside vaccines currently in clinical trials, but none have been licensed for use in humans except mRNA vaccines, which were granted EUA in early 2021.

## EUA for COVID-19 vaccines, in vitro diagnostics (IVDs), and safety concerns

At the onset of the pandemic, a major problem in monitoring and managing the spread of the pandemic was the lack of efficient diagnostic resources, often typified by false results, consequently resulting in risks of delayed or lack of supportive care, exposure of the healthy populace to the disease and possible adverse drug reactions in the case of false positive outcomes. On this premise, the United States FDA adopted EUA to several diagnostic tests for COVID-19. So far, nucleic acid testing for detection of SARS-CoV-2 RNA using the RT-PCR, and serological assays for detection of SARS-CoV-2 specific IgG and IgM antibodies are the two main types of diagnostics with EUA approval for COVID-19. Wang et al. profiled different FDA-EUA approved molecular diagnostics with their sensitivity-specificity ratios. The Centers for Disease Control and Prevention, QIAGEN, PerkinElmer, Thermo Fisher Scientific, and Roche demonstrated a sensitivity-specificity ratio of 1.0, whereas Abbott's Alinity m and BD's BioGx SARS CoV-2 assays depicted sensitivity-specificity ratios of 100%/96.5% and 95%/100%, respectively.^78^ Despite the impressive indices, these assays still require highly trained professionals to deliver an extremely low error margin. In a similar vein, the authors reported a comparative review of the FDA-EUA serological assays as: Autobio Diagnostics (99%/99.04%), Cellex (93.8%/96%), Orth clinical diagnostics (100%/100%), DiaSorin (97.56%/99.3%), Abbott (100%/99.63%), Bio-Rad (92.2%/99.6%), Roche (100%/99.81%), and Siemens Healthcare assay (100%/99.8%). The likelihood ratios were not reported for both assays, but the likelihood ratios of the IVDs might be needless at this stage since most assays are basically qualitative and the urgency for effective therapeutics overshadows the need for viral load determination or antibody titers in an infected patient. Current evidence suggests that clinicians’ judgment should be based on a combination of both RT-PCR and serological assays for COVID-19 diagnosis^79^ although the cost implications for low-resource communities can be a motivation for the development of cheap, affordable, and user-friendly point-of-care IVDs.

As shown in Table 1, there is an avalanche of clinical trials evaluating many investigational COVID-19 vaccines with the aim to provide safe and effective vaccines. The urgency to return to normalcy has inadvertently informed the EUA of some vaccines that are currently premised to establish a herd immunity. No sooner had the EUA of the first mRNA vaccine been given, than the Advisory Committee on Immunization Practices following the Evidence to Recommendation guideline also issued interim recommendations for use in persons aged ≥18 years for the prevention of COVID-19.^80^ Although the first viral vector vaccine, which demonstrated 82% efficacy after a second dose, was approved for emergency supply in the UK,^81^ the exigency of curbing the disease has called for other vaccine strategies that can afford a one-dose administration, efficient logistics, low costs, and availability in low-resource communities. While it is highly likely that more vaccine candidates will gain EUA approval, COVID-19 vaccine hesitancy might pose the next major challenge to achieving herd immunity globally.^82^

Thus far, most COVID-19 vaccines have been reported to be minimal reactogenic. The commonest among the population are injection site pain, fatigue, headache, muscle pain, and joint pain, and close clinical monitoring is highly recommended in certain populations.^83^ One trial reported two cases of adverse events by recipients of an mRNA vaccine, facial edema in one recipient with a history of injection dermatological fillers, and intractable nausea and vomiting in another recipient with a history of severe headache with nausea that required hospitalization. By adopting the Brighton Collaboration case definition criteria for adverse events following immunizations, Centers for Disease Control and Prevention received 66 case reports typified by two mRNA doses administered in different instances over a 4-week period following EUA approval, reporting at rates of 2.5–4.7 cases/million.^83^ A randomized study in the UK demonstrated the safety of an EUA viral vector vaccine administered to old-aged participants,^84^ although in recent time there are reports of blood clotting in individuals who have been vaccinated. These claims are yet to be verified. There are also reports of adverse events in recipients with underlining health conditions, thus suggesting the need for such individuals to be vaccinated under close monitoring.

## Conclusion and perspectives

Considering the recent SARS-CoV-2 variants, efficient vaccine design is an ongoing venture, and this review highlights key components that are pertinent to flattening the curve in subsequent post-first wave COVID-19 spikes while also submitting our expert views herein. In context, emerging results from host immune responses to SARS-CoV-2 are certain to enhance our understanding of the variations in disease susceptibility and clinical outcomes and how to deal with potential variants or other zoonotic pathogens best. It is now established that clinical outcomes correlate with host immunity. Another recent update is the role of the glycocalyx (especially the heparan sulfate) in modulating the likelihood of a viral infection through attraction and repulsive interactions. In parallel, in the face of the highly efficacious vaccines currently administered globally is the indistinct “breakthrough SARS-CoV-2 infections” concerns that account for at least 5% non-protection in a fully vaccinated population. While the mechanism is still unclear, we hypothesized with similar reported infections of dengue fever that featured cross-reactivity between antibodies from homologous strains with heterologous DENV serotypes, resulting in devastating events in the naive population as possible cause of potential spikes in COVID-19 cases post-vaccination. A potentially convoluted scenario is the challenge of establishing cross-reactivities between the alpha, beta, gamma, and delta SARS-CoV-2 strains with the immunogenic components of the current vaccines. It, therefore, calls for a renewed approach to vaccine design where all possible SARS-CoV-2 variants of concern and variants of interest are considered in the formulation of vaccines devoid of cross-reactivity tendencies.

Pointedly, the glycan architecture is seldom indexed in vaccine design considering their role in the infection sequence. While glycans are resistant to structural changes, the enzymes for the glycan assembly (transferases and glycosidases) remain key influencers to compositional variations in the SARS-CoV-2 and in turn linked to disease pathogenesis. Although no SARS-CoV-2 glycan-specific enzyme has been reported so far, their elucidation and status as anti-COVID-19 targets will expand our therapeutic options. Analytical advancements to the tune of Cryo-EM microcrystal electron diffraction are yet to be engaged to unambiguously resolve the complex viral glycans. Results from such assays will provide broader insights into the pathophysiological implications and deepen our understanding of host responses to these pathogens. In a similar vein, the objective of COVID-19 diagnosis should also extend to the viral glycans as the target epitopes to detect infections. In transitioning from EUA to FDA-approved IVDs, the development of point-of-care IVDs, which hold merits such as sensitivity, specificity, result reproducibility, quick and low cost will come in handy in the event of pandemics where frontline health workers are either limited or overstretched.

## Acknowledgments

The authors are grateful for the anonymous reviewers.
